# AI for detection, classification and prediction of loss of alignment of distal radius fractures; a systematic review

**DOI:** 10.1007/s00068-024-02557-0

**Published:** 2024-07-09

**Authors:** Koen D. Oude Nijhuis, Lente H. M. Dankelman, Jort P. Wiersma, Britt Barvelink, Frank F.A. IJpma, Michael H. J. Verhofstad, Job N. Doornberg, Joost W. Colaris, Mathieu M.E. Wijffels, Machine Learning Consortium

**Affiliations:** 1 Department of Orthopedic Surgery, Groningen, Groningen University Medical Centre, Groningen, The Netherlands; 2 Department of Surgery, Groningen, University Medical Centre, Groningen, The Netherlands; 3 Trauma Research Unit Department of Surgery, Erasmus MC, University Medical Center Rotterdam, P.O. Box 2040, Rotterdam, 3000 CA The Netherlands; 4 Department of Orthopedic Surgery, Hand and Arm Center, Massachusetts General Hospital, Boston MA, Harvard Medical School, Boston MA, The Netherlands; 5 University Medical Center, Utrecht, The Netherlands; 6 Department of Orthopedics and Sports Medicine, Erasmus University Medical Centre, Rotterdam, The Netherlands; 7 Department of Orthopaedic and Trauma Surgery, Flinders University and Flinders Medical Centre, Adelaide, Australia

**Keywords:** Trauma, Distal radius fractures, Wrist, Artificial intelligencess

## Abstract

**Purpose:**

Early and accurate assessment of distal radius fractures (DRFs) is crucial for optimal prognosis. Identifying fractures likely to lose threshold alignment (instability) in a cast is vital for treatment decisions, yet prediction tools’ accuracy and reliability remain challenging. Artificial intelligence (AI), particularly Convolutional Neural Networks (CNNs), can evaluate radiographic images with high performance. This systematic review aims to summarize studies utilizing CNNs to detect, classify, or predict loss of threshold alignment of DRFs.

**Methods:**

A literature search was performed according to the PRISMA. Studies were eligible when the use of AI for the detection, classification, or prediction of loss of threshold alignment was analyzed. Quality assessment was done with a modified version of the methodologic index for non-randomized studies (MINORS).

**Results:**

Of the 576 identified studies, 15 were included. On fracture detection, studies reported sensitivity and specificity ranging from 80 to 99% and 73–100%, respectively; the AUC ranged from 0.87 to 0.99; the accuracy varied from 82 to 99%. The accuracy of fracture classification ranged from 60 to 81% and the AUC from 0.59 to 0.84. No studies focused on predicting loss of thresholds alignement of DRFs.

**Conclusion:**

AI models for DRF detection show promising performance, indicating the potential of algorithms to assist clinicians in the assessment of radiographs. In addition, AI models showed similar performance compared to clinicians. No algorithms for predicting the loss of threshold alignment were identified in our literature search despite the clinical relevance of such algorithms.

**Supplementary Information:**

The online version contains supplementary material available at 10.1007/s00068-024-02557-0.

## Introduction

The use of Artificial Intelligence (AI) to perfectly detect and classify fractures on radiographic images and to predict the best treatment option is considered a holy grail. This is also true for distal radius fractures (DRFs), where surgery aims to prevent losing threshold alignment (also known as a fracture being ‘unstable’) after closed reduction. The terminology might be confusing, as “fracture instability” and “fracture redisplacement” are often used interchangeably with “loss of threshold fracture alignment”; they are, however, insufficient and should be avoided where possible.

Detection of DRFs is most often not an issue, but non-displaced fractures or more subtle fracture lines, such as a radial styloid fracture, can be missed [1]. It has been noted that four out of five diagnostic errors made in the emergency department are missed fractures, and about 13–17% of missed fractures are located in the wrist [2, 3]. AI could be of great help here in aiding physicians.

DRF classification should (1) enable a standardized method to describe fractures and give guidance in the proper treatment per classification, (2) provide a consistent method of recording in the electronic patient system for evaluation of the patient in research, and (3) help compare studies using the same classifications and therefore optimize the treatment protocols. Considering this, a reliable fracture classification system can provide insight into clinical decision-making [4]. Therefore, a fracture classification tool without inherent surgeon bias is of interest.

When a DRF is displaced, closed reduction and cast immobilization are traditionally chosen [5]. However, secondary displacement occurs in up to 64% of the patients [6]. Identifying fractures likely to lose threshold alignment could greatly help clinical decision-making between nonoperative and surgical treatment. However, the accuracy and reliability of current fracture loss of threshold alignment prediction tools still need to be improved [7–10].

AI can execute tasks that humans previously performed. Specifically, Convolutional Neural Networks (CNN), which can evaluate visual input, have been of interest [11]. While earlier AI methods have led to applications with subhuman performance, recent CNNs can match and even surpass the capacity of humans to detect certain fractures on radiographs, focusing on isolated fracture types per model [12–16]. The strength of computers and algorithms is their ability to perform many calculations rapidly, consistently and without exhaustion. CNNs can be used to implement automated fracture detection, classification, and prediction algorithms to guide clinicians in clinical and emergency settings. There has been less focus on using CNNs as a prediction tool, even though this might be the most valuable attribution for treatment decisions. Given the above-mentioned challenges within the care for DRFs and the promising development of AI, we conducted a systematic review to give an overview of studies using CNNs with radiographs to detect, classify, and/or predict loss of threshold alignment of DRFs. This study aimed to answer two questions: (1) What is the accuracy of current CNNs in detecting and classifying DRFs and predicting their loss of threshold alignment on radiographs? (2) Does the use of CNNs outperform the diagnostic performance of clinicians?

## Methods

### Article selection, quality assessment, and data extraction

The systematic literature search was performed according to the PRISMA statement [17] and conducted in Medline ALL, Embase, Web of Science Core Collection, Cochrane Central Register of Controlled Trials and Google Scholar (100 top-ranked) in January 2024. The search strategy can be found in Appendix 1. This review was not registered online.

After removing duplicities, two authors (LHMD and KDON) independently screened the title and abstract for potential inclusion. Subsequently, a full-text review was done on the remaining articles with the defined inclusion and exclusion criteria. Articles were included if they described the use of CNNs to detect or classify DRFs or to predict loss of threshold alignment of DRFs on plain radiographs. Papers describing studies in children, reviews, letters, conference abstracts, surgical techniques, studies using robots, animal and cadaveric studies, non-orthopaedic fractures, and studies not published in English or Dutch were excluded. The inconclusive inclusion of articles was discussed afterward by the two reviewers. Covidence (Veritas Health Innovation, Melbourne, Australia) was used for the screening process and full-text review.

To assess the quality of the included articles, two reviewers (KDON, JW) independently used a modified version of the methodologic index for non-randomized studies (MINORS). A third reviewer was consulted if the scoring was inconsistent (LHMD). Studies with low scores on three or more items were excluded. Standardized forms were used to extract and record data (Microsoft Excel Version 16.21; Microsoft Inc, Redmond, WA, USA).

### Outcome measures

The primary outcome was the performance of the AI model used, given in sensitivity, specificity, accuracy, Area Under the Receiver Operator Characteristics Curve (AUC), F-1 score, and average precision when present. The secondary outcome was comparing the AI models’ performance to clinicians’ performance. The highest possible F1-score is 1.0, indicating perfect precision and recall, and the lowest possible value is 0. The AUC is a score to measure the ability of a classifier to distinguish between classes. Scores lie between 0.5 (classifier equal to chance) and 1 (a perfect classifier), scores < 0.5 are not reported as they predict the wrong result. Average precision 50 (AP50) is a metric for localizing objects, meaning there is a 50% overlap between the object predicted by the algorithm versus the golden standard.

From each included article, the following data points were collected: author, year of publication, type of CNN model used, radiographic views, output classes, ground truth label assignment, number of patients or radiographs, performance metric (e.g. sensitivity, specificity, accuracy), comparison of CNN versus radiologist or reports, whether external validation was performed and potential open access availability of the model (Table 1). The reported output classes include DRF detection (fracture yes/no), localization and classification.

**Table 1:** **Description of studies**

Author, year	Study type	AI Models used (Type)	Projection of radiograph	Output classes	Ground truth label assignement	Number of radiographs (number of fractures)	External validation	Performance metics	Perfomance outcomes	Comnparison CNN vs. radiologist	Open access
**Antilla et al., 2022**	Detection	DL: U-Net	PA and lateral	Two (fracture yes/no)	3 hand surgeons	*Trained on*: 6948 *Tested on*: 772 (271)	No	*With cast:* Sensitivity Specificity AUC Accuracy *Without cast:* Sensitivity Specificity AUC Accuracy	92% (90–94%) 88% (84–92%) 0.96 (0.94–0.97) 91% (89–93%) 86% (81–91%) 89% (84–93%) 0.94 (0.91–0.96) 88% (85–91%)	No	Yes, contact corresponding author
**Blüthgen et al., 2020**	Detection and localization	DL: ViDi Suite Version 2.0	PA and lateral	Two (fracture yes/no)	2 radiology residents	*Trained on*: 524 (166) *Tested on*: Internal: 100 (42) External: 200 (100)	Yes	**Detection** *Internal dataset (model1; model2)* Sensitivity Specificity AUC *External dataset (model1; model2)* Sensitivity Specificity: AUC: **Localization** *Internal dataset: (AP, LAT, Combined views)* Model 1 Model 2 *External dataset: (AP, LAT, Combined views)* Model 1 Model 2	81% (58–95%); 90% (70–99%) 100% (88–100%); 97% (82–100%) 0.95 (0.85–0.99); 0.96 (0.87–1.00) 80% (66–90%); 82% (69–91%) 86% (73–94%); 78% (64–88%) 0.87 (0.79–0.93) 0.89 (0.81–0.94) 100%, 88%, 94% 94%, 87%, 89% 91%, 92%, 88% 100%, 89%, 93%	Yes	No
**Cohen et al., 2022**	Detection	CNN: Boneview	AP, oblique and specific views of the carpus	Two (fracture yes/no)	3 senior musculoskeletal radiologists	*Trainded on*: 1342 *Validated: 192* *Tested on*: 383 (166)	Yes	*All wrist fractures* Sensitivity Specificity *Distal radius (166 fracturen)* sensitivity	83% (78–87%) 96% (93–97%) 89%	Yes	No
**Gan et al., 2019**	Detection	CNN: inception-v4	AP	Two (fracture yes/no)	3 senior orthopedists	*Trained on*: 2040 (1491) *Tested on*: 300 (150)	No	Sensitivity Specificity AUC Accuracy Youden index	90% (85–95%) 96% (93–99%) 0.96 93% (90–96%) 0.86 (0.80–0.91)	Yes	No
**Hardalac et al., 2022**	Detection	CNN: WFD-C	N.A.	Two (fracture yes/no)	1 radiologist and 2 orthopedists.	*Trained on*: 434 (all) *Validated on*: 54 (all) *Tested on*: 54 (all)	No	AP50	86.39	No	Yes, through Github
**Joshi et al., 2022**	Detection and Localization	CNN: mask R-CNN	N.A.	Two (fracture yes/no)	Multiple orthopaedic surgeons and radiologist.	*Trained on*: 221 (all) *Validated on*: 63 (all) *Tested on*: 32 (all)	No	*Fracture detection:* AP50 AP75 *Fracture segmentation:* AP50 AP75	92.278 79.003 77.445 52.156	No	No
**Kim et al., 2021**	Detection	CNN: DenseNet-161 and ResNet-152	AP and bilateral oblique	Two (fracture yes/no)	Radiological reports	*Trained on*: 8994 (4551) *Tested on*: 990 (300)	No	*Densenet-161:* Sensitivity Specificity AUC Accuracy *ResNet-152* Sensitivity Specificity AUC Accuracy	90.3% ±1.4 90.3% ±1.3 0.962 90.3% ±1.3 88.6% ±1.0 88.4% ±1.0 0.947 88.5% ±1.0	No	No
**Kim et al., 2018**	Detection	CNN: inception-v3	Lateral	Two (fracture yes/no)	1 radiology registrar	*Trained on*: 1111 (695) *Validated on*: 139 *Tested on*: 139 *Extra test set*: 100	Yes	*External dataset:* Sensitivity Specificity AUC	90% 88% 0.954	No	No
**Lee et al., 2023**	Detection	DL: DeepLab v3 and NasNet	AP, Lateral, Oblique	Two (fracture yes/no)	1 orthopedic surgeon, 1 muscoskeletal radiologist	*Trained on: 3032* *Tested on: 758* *External validation: (332)*	No	*Interal dataset*: Sensitivity Specificity Accuracy AUC	97.2% (95.6–99%) 83.2% (80.7–95.7%) 87.2% (85.2–89%) 0.903 (0.887–0.918)	Yes	No
**Lindsey et al., 2018**	Detection	CNN	PA and lateral	Two (fracture yes/no)	Multiple orthopedic surgeons.	*Trained on*: 31,490 (NA) *Validated on*: 1400 *Internal test*: 3500 *External test*:1400	Yes	*Internal test:* AUC *External test:* AUC *Clinician dataset:* Sensitivity Specificity AUC	0.967 (0.960–0.973) 0.975 (0.965–0.982) 93.9% (83.2–98.0) 94.5% (90.6–97.2) 0.990 (0.971–0.997)	Yes	No
**Min et al., 2023**	Localisation and classification	DL: YOLOv5	PA	Location fracture. Extra- vs. intra-articular fracture	Location: medical student. Classification: 3 orthopedic registrars	Trained on: 334 (292) *Tested on: 66 (57)*	No	*Localisation*: Average IoU *Classification*: AUC Accuracy Sensitivity Specificity F1-score	0.816 ± 0.071 0.82 81% 83% 73% 0.86	No	No
**Oka et al., 2021**	Detection	CNN: VGG16	AP and lateral	Two (fracture yes/no)	Clinical diagnosis orthopedic surgeons	*Trained on*: 743 (569) *Validated on*: 120 (80) *Tested on*: 120 (80)	No	Sensitivity Specificity Accuracy AUC	98.6% ± 1.8 96.7% ± 3.5 98.0% ± 1.6 0.991 (0.984–0.999)	No	No
**Raisuddin et al., 2021**	Detection	DL: DeepWrist, Gradcam	PA and lateral	Two (fracture yes/no)	2 radiologists independently.	*Trained on*: 3873 (953) *Tested On: * Trivial cases: 414 Hard cases: 210	No	*Trivial cases * Sensitivity Specificity AUC F1-score *Hard cases* Sensitivity Specificity AUC: Balanced accuracy F1-score *Combination trivial hard:* AUC	97% (94–100%) 87% (79–93%) 0.99 (0.98–0.99) 0.95 (0.92–0.97) 60% (40–80%) 92% (87-0.97%) 84% (72–93%) 0.76 (0.65–0.87) 0.63 (0.44–0.80) 0.97 (0.95–0.98)	Yes	Yes, through Github
**Suzuki et al., 2022**	Detection	CNN: EfficientNet B2 - EfficientNet B5	AP and lateral	Two (fracture yes/no)	2 orthopedic surgeons.	*Trained on*: 1333 (722) *Tested on*: 300 (150)	No	Sensitivity Specificity AUC Accuracy	98.7% (92.8–99.8%) 100% (95.1–100%) 0.993 (0.949–0.997) 99.3% (96.3–99.9%)	Yes	No
**Thian et al., 2019**	Detection	CNN: Inception-ResNet Faster R-CNN	AP and lateral	Two (fracture yes/no)	2 radiologists	*Trained on*: 13,153 (2130) *Validated on*: 1461(341) *External testset*: 1048	Yes	*Per study (AP-LAT combined):* Sensitivity Specificity AUC	98.1% (95.6–99.4%) 72.9% (67.1–78.2%) 0.895 (0.870–0.920)	No	No
**Tobler et al., 2021**	Detection and classification	CNN: ResNet18	Frontal and lateral	Two (fracture yes/no)	2 musculoskeletal senior radiologists	*Trained on*: 7997 (3656) *Tested on*: Set A: 582 Set B: 326	Yes	*Detection (set A; set B):* AUC Accuracy *Fragment displacement (set A; set B)*: AUC Accuracy *Joint involvement (set A; set B):* AUC Accuracy *Multiple fractures (set A; set B)*: AUC Accuracy	0.975 (0.957–0.992); 0.983 93.8% 0.589 (0.463–0.715); 0.916 59.7% 0.618 (0.516–0.720); 0.898 63.7% 0.842 (0.774–0.911); 0.905 78.2%	Yes	No
**Ureten et al., 2022**	Detection	CNN: Resnet-50, VGG-16, Googlenet	N.A.	Two (fracture yes/no)	1 orthopedic surgeon and 1 radiologist	*Trained /validated on*: 410 (275) *Tested on*: 135	No	*VGG-16; ResNet-50; GoogLeNet* Sensitivity Specificity Accuracy Precision	96.8%; 94.9%; 90.6% 90.3%; 84.2%; 85.9% 93.3%; 88.9%; 88.1% 89.7%; 82.4%; 85.3%	No	No
**Zhang et al., 2023**	Detection	DL: Ensemble model	AP and lateral	Two (fracture yes/no)	1 orthopedist and 1 radiologist	*Trained on*: 4579 (2268) *Validated on*: 979 (486) *Tested on*: 978 (486)	No	Accuracy Sensitivity Specificity	97.0% (95.71–97.96%) 95.7% (93.44–97.13%) 98.4% (96.73–99.18%)	No	No

N.A.: Not assesed AI: Artificial intelligence. DSS: decision support systems. CNN: Convolutional Neural Networks. DL: deel learning. AUC: Area Under the Curve. AP50/AP75: Average precision 50/75. ResNet: Residual network. VGG: Visual geometry group. WFD-C: wrist fracture detection-combo. PA: Posterior-Anterior. AP: Anterior-Posterior. LAT: Lateral. ±: standard deviation. IoU: intersection over union (average overlap)

### Quality appraisal

In this study, the MINOR Criteria included the following items: disclosure, input features, ground truth, external validation, performance metric, and AI model (Table 2). Disclosure was reported in almost all the studies except Suzuki et al. [18]. All studies clearly described the study aim. Eight studies did not describe the input features used [15, 19–25]. Five studies [19, 22–25] did not specify the ground truth used as a reference standard for the AI model. The external validation method was described only in six studies [13, 15, 26–29]. Two studies [23, 24] did not describe the performance metric assessed in the studies. All studies described which AI model was used. According to the outcomes of the MINORS criteria, five studies were excluded because three or more criteria were missing.

**Table 2:** Quality assessment according to adapted MINORS criteria

Study type	Author, year	Disclosure	Study aim	Input features	Ground truth	External validation method	Performance metric	AI model
Detection	**Antilla et al., 2022**	1	1	1	1	0	1	1
Detection and localisation	**Blüthgen et al., 2020**	1	1	1	1	1	1	1
Detection	**Cohen et al., 2022**	1	1	1	1	1	1	1
Detection	**Ebsim et al., 2019**	1	1	0	0	0	1	1
Detection and localisation	**Yahalomi et al., 2018**	1	1	0	0	0	0	1
Classification	**Yang et al., 2021**	1	1	0	0	0	0	1
Detection	**Gan et al., 2019**	1	1	1	1	0	1	1
Localisation	**Hardalac et al., 2022**	1	1	0	1	0	1	1
Detection	**Javed et al., 2023**	1	1	0	0	0	1	1
Detection	**Joshi., 2022**	1	1	0	1	0	1	1
Detection	**Kim, 2018**	1	1	1	1	0	1	1
Detection	**Kim et al., 2021**	1	1	1	1	1	1	1
Detection	**Lee et al., 2023**	1	1	1	1	0	1	1
Detection	**Lindsey et al., 2018**	1	1	0	1	1	1	1
Localisation and classification	**Min et al., 2023**	1	1	1	1	0	1	1
Detection	**Oka et al., 2021**	1	1	1	1	0	1	1
Detection	**Raisuddin et al., 2021**	1	1	1	1	0	1	1
Detection	**Rashid et al., 2023**	1	1	0	0	0	1	1
Detection	**Suzuki et al., 2022**	0	1	1	1	0	1	1
Detection	**Thian et al., 2019**	1	1	1	1	1	1	1
Detection and classification	**Tobler et al., 2021**	1	1	1	1	1	1	1
Detection	**Ureten et al., 2022**	1	1	1	1	0	1	1
Detection	**Zhang et al. 2023**	1	1	1	1	0	1	1

### Statistical analysis

If possible, a meta-analysis will be performed. If not possible due to the variance in utilized algorithms, an overview will be given, describing the number of patients or radiographs used in training and (internal or external) validation, accuracy, sensitivity, specificity, AUC, F-1 score, average precision, and Youden index when present.

## Results

### Included studies

**Fig. 1:**
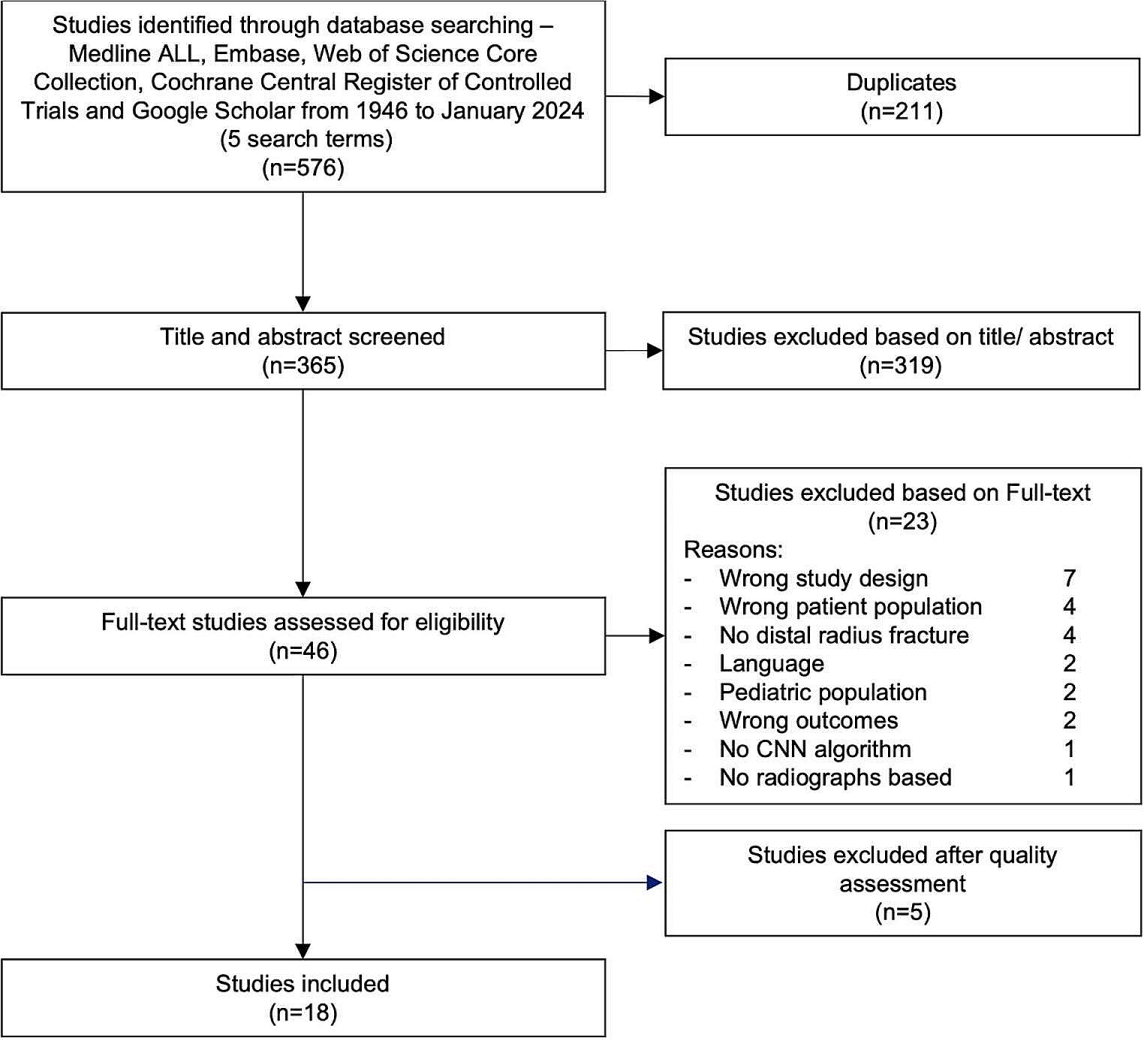
Inclusion and exclusion flowchart

The literature search resulted in a total of 576 articles; after removal of duplicates, 365 abstracts were screened. Forty-six studies were full-text screened, and after quality assessment, eighteen studies were included in this review. (Fig. 1). No new eligible studies were identified through reference lists.

## Description of studies

Of the included studies, fourteen studies described detection [15, 18, 20, 26, 28, 30–38], one study both detection and classification [29], two studies both detection and localization [13, 21] and one study localization and classification [39] of DRFs. No studies on the prediction of loss of threshold alignment were found. Four studies used posterior-anterior (PA) and lateral radiographs [15, 32, 36, 40], in five studies anterior-posterior (AP) and lateral [18, 28–30, 38], and in three studies [26, 34, 37] an extra oblique projection was used. Three studies only used lateral [33], AP [31], or PA [39] radiographs, and in three studies [20, 21, 35], the projection was not clearly described. As the ground truth, fifteen [15, 18, 20, 21, 26, 28, 29, 31–33, 36–40] studies used one or more radiologists’ or surgeons’ expertise to detect DRF. In addition, one study [34] used the radiological reports, checked and verified by a radiology registrar competent, and one study [30] used the clinical diagnosis of orthopaedic surgeons. In one study [35], the ground truth was not reported. The number of included radiographs ranged from 221 [21] to 31,490 [15] and from 32 [21] to 3500 [15] for training and testing sets, respectively. Validation sets were used in six studies [15, 20, 21, 26, 28, 30], ranging from 54 [20] to 1461 [28] radiographs. The total number of fractures on the radiographs used in the studies ranged from 221 [21] to 4452 [34] DRFs.

### Detection

The sensitivity of fracture detection was reported in fourteen studies [15, 18, 22, 26, 28, 30–35, 37, 38, 40], ranging from 80% [13] to 99% [18]. Specificity was also reported, from 73% [28] to 100% [13, 18]. The AUC was reported in twelve studies [15, 18, 27, 28, 30–33, 36, 37, 40, 29] ranging from 0.87 [13] to 0.99 [30]. The accuracy was reported in nine studies [18, 29–32, 34, 35, 37, 38] ranging from 82% [22] to 99% [18]. In addition, Raisuddin et al. [36] reported a balanced accuracy of 76%. See Table 1.

Two CNN models were compared by Kim et al. [34], where the sensitivity, specificity, AUC and accuracy were similar for both models. Lindsey et al. [15] reported the performance of different test sets separately, where the AUC was 0.97, 0.98, and 0.99 for the internal, external, and clinical data test sets, respectively.

### Classification

Two studies reported the performance of the classification of DRFs [29, 39]. The AUC assessed separately by Tobler et al. [29] on fragment displacement, joint involvement, and detection of multiple fractures was 0.59, 0.68, and 0.84, respectively. The accuracy was 60%, 64% and 78%, respectively [29]. Min et al. reported an AUC of 0.82, accuracy of 81%, sensitivity of 83%, specificity of 72% and a F1-score of 0.86.

### AI versus clinicians

Among the included studies, eight [15, 18, 26, 29, 31, 36, 37, 40] compared the performance of AI and clinicians’ performance. According to Blüthgen et al. [40], radiologists’ performance was comparable to internal data and better on external data. Cohen et al. [26] found AI sensitivity significantly higher than initial radiology reports (IRR), with combined AI and IRR showing even greater sensitivity. Gan et al. [31] demonstrated that AI outperforms radiologists in accuracy, sensitivity, specificity, and Youden index. Comparisons with orthopaedic surgeons showed similar results. Lindsey et al. [15] revealed comparable sensitivity and AUC of aided and unaided emergency medicine clinicians by CNN. Notably, the model showed higher specificity compared to unaided clinicians. Raisuddin et al. [36] showed higher radiologist performance in normal cases and similar performance in hard cases.

Suzuki et al. [18] showed equal to better accuracy, sensitivity and specificity of CNN versus orthopaedic surgeons, though without statistically significant differences.

In Lee et al. [37], the sensitivity, specificity, accuracy, and AUC of two reviewers aided by AI increased in all fields compared to unaided. In addition, this study showed a decrease in mean interpretation time when aided by AI. Lastly, Tobler et al. [29] reported higher AUC for radiology residents than AI’s assessment of DRFs without osteosynthetic material or cast.

## Discussion

This systematic review provides an overview of various computer vision algorithms for detecting and classifying DRFs on plain radiographs. Overall, the included studies showed that the performance of DRF detection is excellent, with accuracies and AUC up to 100% and 0.99, respectively. Compared with clinicians’ performance, AI had at least comparable and often better results. The development of a DRF classification model of DRF reported accuracies and AUC of 60–81% and 0.59–0.84, respectively [29, 39]. No studies describing algorithms predicting the loss of threshold alignment of DRFs were found.

This current study has several limitations. First, the comparability of the studies was limited. The studies were not consistent in the reported performance metrics. In addition, the studies used various types of DL and CNN models. However, the results of the studies show comparable performances of the different types of AI used, and the heterogenicity of the models did not affect our research questions. Secondly, the role of AI in the classification of DRF was only reported in two studies with different assessments of classifications. Therefore, evaluating AI’s overall ability to classify DRFs is difficult. Thirdly, the ground truth was differently defined between studies or even not reported at all. Lastly, only six out of 18 studies performed an external validation of the AI model. To use AI in clinical practice, a model must be trained, tested, externally validated, and preferably prospectively validated. This validation is crucial to explore transportability and bias [41]. The lack of commonplace external validation shows that most algorithms cannot be used for daily practice yet.

The strengths of this review include the broad search in different databases and the quality assessment according to the modified MINORS criteria with AI-specific factors.

The included studies reported a sensitivity and specificity between 80 and 100% in detecting DRFs. There was a significant decrease in performance between the internal and external validation set on the separate assessment of the performance on AP and lateral views. This showed the necessity of training a DL model on data comparable to the intended target data. On the other hand, to eventually build a model capable of being used on an outside institution, further improving the AI model’s performance on external validation data sets is necessary. When AP and lateral views were combined, they showed similar performance on both internal and external sets. The reported AUC and accuracy were good to excellent across the included studies. The F1-score reported in the included studies showed poor to good precision.

**Fig. 2:**
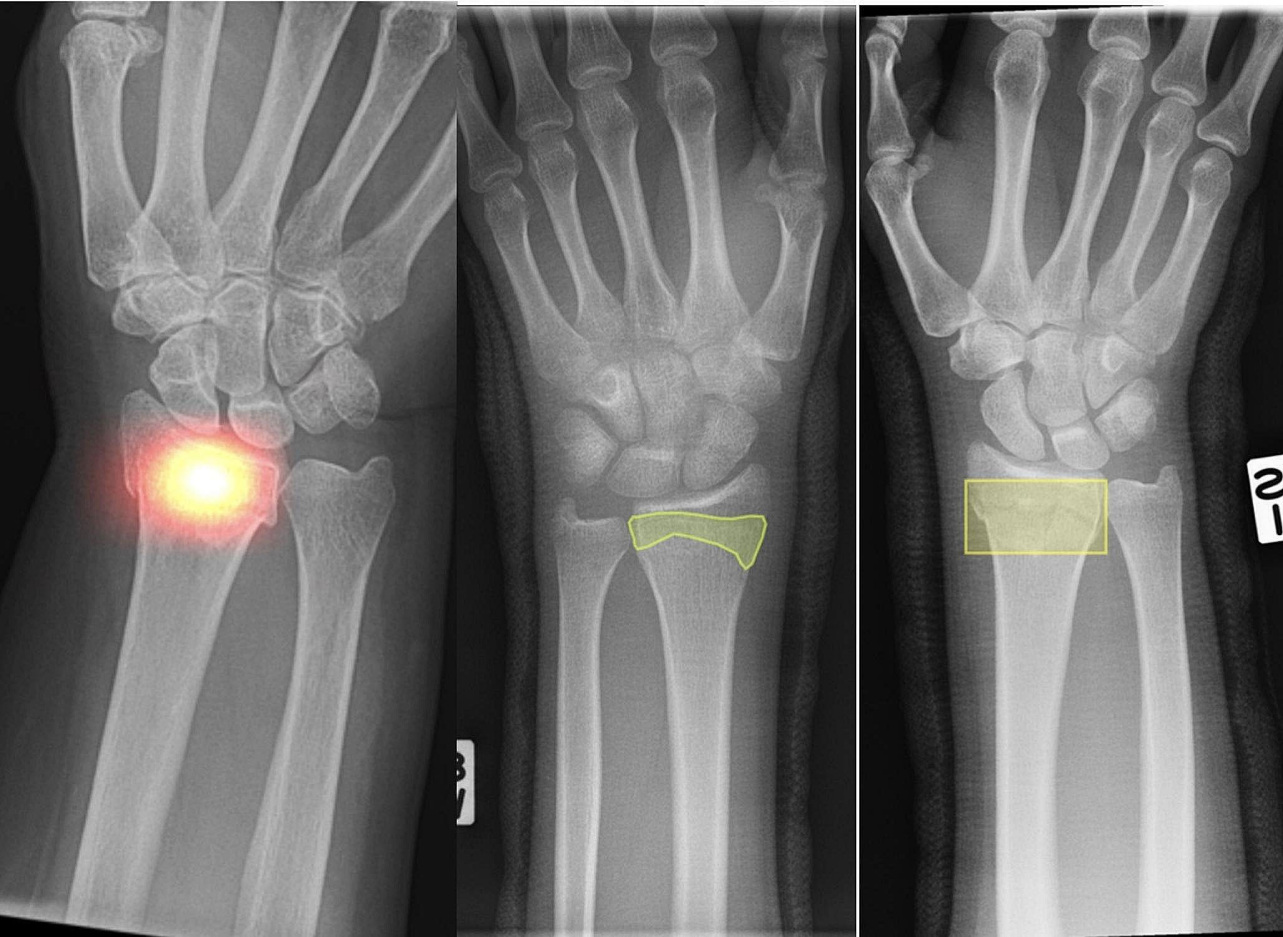
Different visualization of localization of fractures on PA radiographs. From left to right: a heatmap, a polygon and a bounding box

Three studies used localization in addition to detection. This helps clinicians look into the black box of the algorithm, allowing them to check for any mistakes the algorithm might make. See Fig. 2 for different options for presenting localisations. Future studies might choose to implement similar visualizations to help clinicians implement this in their daily practice.

Some of the included studies used the same CNN architecture backbone. For instance, Inception version 3 and version 4 were used in two studies [31, 33], both show comparable sensitivity, specificity, and AUC. In addition, one study [28] used a combined Inception- Resnet-Faster R-CNN and showed lower specificity and AUC. The ResNet algorithm or backbone was used in five studies [21, 27–29, 35], all showing comparable performances of the algorithms.

In conclusion, AI models for detecting DRFs demonstrate promising performances across various metrics. However, results may vary depending on each study’s dataset, model architecture, and evaluation methods. From a clinical perspective, DL and CNN algorithms have the potential to aid clinicians in medical imaging tasks and improve diagnostic accuracy in recognizing and consistently recording DRFs. Furthermore, we recommend focusing on diligent AI research, which involves presenting extensive outcomes, a comprehensive explanation of the dataset and the ground truth, and proper external validation.

## Electronic supplementary material

Below is the link to the electronic supplementary material.

Supplementary Material 1

## Data Availability

No datasets were generated or analysed during the current study.
