# Supplementary material for: AI for detection, classification and prediction of loss of alignment of distal radius fractures; a systematic review
Source: Eur J Trauma Emerg Surg. 2024 Jul 9;50(6):2819–31. doi: 10.1007/s00068-024-02557-0 (PMC11666746; doi:10.1007/s00068-024-02557-0)
Supplement: Supplementary file 1 — Supplementary Material 1 [file 68_2024_2557_MOESM1_ESM.docx]

**Appendix 1.**

| **Database searched** | **Platform** | **Years of coverage** | **Records** | **Records after duplicates removed** |
| --- | --- | --- | --- | --- |
| Medline ALL | Ovid | 1946 - Present | 124 | 112 |
| Embase | Embase.com | 1971 - Present | 213 | 102 |
| Web of Science Core Collection* | Web of Knowledge | 1975 - Present | 134 | 58 |
| Cochrane Central Register of Controlled Trials** | Wiley | 1992 - Present | 5 | 3 |
| Additional Search Engines: Google Scholar*** (100 top-ranked) | | | 100 | 91 |
| **Total** | | | **576** | **365** |

*Science Citation Index Expanded (1975-present) ; Social Sciences Citation Index (1975-present) ; Arts & Humanities Citation Index (1975-present) ; Conference Proceedings Citation Index- Science (1990-present) ; Conference Proceedings Citation Index- Social Science & Humanities (1990-present) ; Emerging Sources Citation Index (2005-present)

***Google Scholar was searched via "Publish or Perish" to download the results in EndNote.

No other database limits were used than those specified in the search strategies

**Embase 213**

('distal radius fracture'/exp OR 'wrist fracture'/de OR 'radius fracture'/de OR (((distal) NEAR/6 (radius OR radial*) NEAR/6 (fractur* OR broken)) OR ((Barton OR Colles OR Smith OR chauffeur OR Hutchinson OR wrist OR radius OR radial OR colles*) NEAR/3 (fractur* OR broken)) OR ((wrist) NEAR/3 (radiograph* OR X-ray))):ab,ti,kw) **AND** ('artificial intelligence'/exp OR 'machine learning'/exp OR 'artificial intelligence software'/de OR (algorithm/exp AND 'prediction and forecasting'/exp) OR (((artificial* OR machine) NEAR/3 (intelligen*)) OR ((machin* OR deep) NEAR/3 (learning*)) OR ((neural) NEAR/3 (network*)) OR AI OR ((algorithm* OR computer-assist* OR computer-aid*) NEAR/3 (predict*))):ab,ti,kw)

**Medline 124**

(Wrist Fractures/ OR exp Radius Fractures/ OR (((distal) ADJ6 (radius OR radial*) ADJ6 (fractur* OR broken)) OR ((Barton OR Colles OR Smith OR chauffeur OR Hutchinson OR wrist OR radius OR radial OR colles*) ADJ3 (fractur* OR broken)) OR ((wrist) ADJ3 (radiograph* OR X-ray))).ab,ti,kf.) **AND** (exp Artificial Intelligence/ OR (exp Algorithms/ AND exp Forecasting/) OR (((artificial* OR machine) ADJ3 (intelligen*)) OR ((machin* OR deep) ADJ3 (learning*)) OR ((neural) ADJ3 (network*)) OR AI OR ((algorithm* OR computer-assist* OR computer-aid*) ADJ3 (predict*))).ab,ti,kf.)

**Cochrane 5**

((((distal) NEAR/6 (radius OR radial*) NEAR/6 (fractur* OR broken)) OR ((Barton OR Colles OR Smith OR chauffeur OR Hutchinson OR wrist OR radius OR radial OR colles*) NEAR/3 (fractur* OR broken)) OR ((wrist) NEAR/3 (radiograph* OR X-ray))):ab,ti) **AND** ((((artificial* OR machine) NEAR/3 (intelligen*)) OR ((machin* OR deep) NEAR/3 (learning*)) OR ((neural) NEAR/3 (network*)) OR AI OR ((algorithm* OR computer-assist* OR computer-aid*) NEAR/3 (predict*))):ab,ti)

**Web of Science 134**

TS=(((((distal) NEAR/5 (radius OR radial*) NEAR/5 (fractur* OR broken)) OR ((Barton OR Colles OR Smith OR chauffeur OR Hutchinson OR wrist OR radius OR radial OR colles*) NEAR/2 (fractur* OR broken)) OR ((wrist) NEAR/2 (radiograph* OR X-ray)))) **AND** ((((artificial* OR machine) NEAR/2 (intelligen*)) OR ((machin* OR deep) NEAR/2 (learning*)) OR ((neural) NEAR/2 (network*)) OR AI OR ((algorithm* OR computer-assist* OR computer-aid*) NEAR/2 (predict*)))))

**Google Scholar 100**

"radius|radial|Barton|Colles|Smith|chauffeur|Hutchinson|wrist|colles fracture|fractures"|"wrist radiography" "artificial|machine intelligence"|AI|"machine|deep learning"|"neural network"

'radius|radial|Barton|Colles|Smith|chauffeur|Hutchinson|wrist|colles fracture|fractures'|'wrist radiography' 'artificial|machine intelligence'|AI|'machine|deep learning'|'neural network'
